# Increased oxidative phosphorylation through pyruvate dehydrogenase kinase 2 deficiency ameliorates cartilage degradation in mice with surgically induced osteoarthritis

**DOI:** 10.1038/s12276-025-01400-9

**Published:** 2025-02-03

**Authors:** Jin Han, Yoon Hee Kim, Seungwoo Han

**Affiliations:** 1 Laboratory for Arthritis and Cartilage Biology, Research Institute of Aging and Metabolism, Kyungpook National University, Daegu, Republic of Korea; 2 Cell & Matrix Research Institute, School of Medicine, Kyungpook National University, Daegu, Republic of Korea; 3 Division of Rheumatology, Department of Internal Medicine, School of Medicine, Kyungpook National University, Daegu, Republic of Korea

**Keywords:** Translational research, Cartilage

## Abstract

Chondrocytes can shift their metabolism to oxidative phosphorylation (OxPhos) in the early stages of osteoarthritis (OA), but as the disease progresses, this metabolic adaptation becomes limited and eventually fails, leading to mitochondrial dysfunction and oxidative stress. Here we investigated whether enhancing OxPhos through the inhibition of pyruvate dehydrogenase kinase (PDK) 2 affects the metabolic flexibility of chondrocytes and cartilage degeneration in a surgical model of OA. Among the PDK isoforms, PDK2 expression was increased by IL-1β in vitro and in the articular cartilage of the DMM model in vivo, accompanied by an increase in phosphorylated PDH. Mice lacking PDK2 showed significant resistance to cartilage damage and reduced pain behaviors in the DMM model. PDK2 deficiency partially restored OxPhos in IL-1β-treated chondrocytes, leading to increases in APT and the NAD^+^/NADH ratio. These metabolic changes were accompanied by a decrease in reactive oxygen species and senescence in chondrocytes, as well as an increase in the expression of antioxidant proteins such as NRF2 and HO-1 after IL-1β treatment. At the signaling level, PDK2 deficiency reduced p38 signaling and maintained AMPK activation without affecting the JNK, mTOR, AKT and NF-κB pathways. p38 MAPK signaling was critically involved in reactive oxygen species production under glycolysis-dominant conditions in chondrocytes. Our study provides a proof of concept for PDK2-mediated metabolic reprogramming toward OxPhos as a new therapeutic strategy for OA.

## Boosting OxPhos in chondrocytes mitigates osteoarthritis progression

Osteoarthritis is a common joint disease where cartilage breaks down, causing pain and stiffness. Chondrocytes, the cells in cartilage, usually rely on glycolysis for energy production. However, in early OA, they can switch to oxidative phosphorylation as an alternative energy pathway. This study aimed to see if modulating chondrocyte metabolism could slow OA progression. Researchers focused on PDK2, a protein that inactivates PDH, thereby reducing OxPhos activity in chondrocytes. They used mice genetically modified to lack PDK2 and compared them with normal mice with surgically induced OA. They found that, without PDK2, chondrocytes had better energy balance by using OxPhos more effectively and less oxidative stress, which slowed OA progression. This suggests that targeting PDK2 could be a new way to treat OA by improving chondrocyte metabolism.

This summary was initially drafted using artificial intelligence, then revised and fact-checked by the author.

## Introduction

Osteoarthritis (OA) is the most common form of degenerative joint disease and is characterized by chondrocyte apoptosis and degradation of the cartilage extracellular matrix (ECM), which ultimately leads to joint failure^1^. Chondrocytes, the only cell type found in cartilage tissues, produce cartilage-specific ECM proteins such as type 2 collagen (Col2) and aggrecan, and they become trapped within the ECM proteins they produce, resembling a state of hibernation^2^. As cartilage is an avascular tissue, chondrocytes exist in hypoxic conditions despite the diffusion of oxygen from synovial fluid or subchondral bone^3^. In such a hypoxic milieu, chondrocytes rely predominantly on glycolysis rather than oxidative phosphorylation (OxPhos), which accounts for less than 10% of total cellular ATP production^4,5^. However, chondrocytes also exhibit a notable level of metabolic flexibility in the catabolic state of early OA, where they can alter their metabolic machinery toward OxPhos^5,6^. This allows them to adapt to catabolic conditions, thereby enhancing their survival and function. As OA progresses and mitochondrial dysfunction occurs^7,8^, however, the metabolic adaptation of OA chondrocytes reaches its limit, leading to a metabolic shift toward the glycolytic pathway^8^. Chondrocytes in early-stage OA of Kellgren and Lawrence grade 1, compared with those in advanced-stage OA of Kellgren and Lawrence grade 4, exhibit a metabolic phenotype characterized by increased OxPhos and decreased glycolysis^9^. This metabolic shift not only makes it increasingly difficult for chondrocytes to meet their energy demands but also amplifies oxidative stress in a lactate dehydrogenase-mediated manner^8^. The increase in oxidative stress, in turn, worsens mitochondrial dysfunction, including increased mitochondrial DNA damage and membrane permeability, eventually contributing to chondrocyte apoptosis and senescence^10,11^. In this way, the glycolysis-prone metabolism of chondrocytes interacts with and amplifies both mitochondrial dysfunction and oxidative stress during OA progression^12^. Despite the understanding that metabolic imbalances in chondrocytes play a crucial role in the pathogenesis of OA, we still lack conclusive evidence on whether redirecting chondrocyte metabolism toward OxPhos could indeed impact its progression.

Glucose that enters chondrocytes mainly through glucose transporter (GLUT) 1 and GLUT3 is metabolized into pyruvate, the end product of glucose catabolism^13^. This pyruvate moves to the mitochondria, where it is converted to acetyl-coenzyme A (acetyl-CoA) by pyruvate dehydrogenase (PDH), the enzyme that links the cytoplasmic glycolysis pathway to the mitochondrial tricarboxylic acid (TCA) cycle. As a canonical input to the TCA cycle, an increase in acetyl-CoA levels leads to an increase in the rate of the TCA cycle and, consequently, OxPhos^14^. Therefore, the activity of PDH, which converts pyruvate into acetyl-CoA, is the overall rate-limiting and gatekeeping factor in pyruvate-driven OxPhos^14^. The activity of PDH is regulated by PDH kinase (PDK), a serine/threonine kinase that phosphorylates the α subunit of PDH, thereby inactivating it^15^. The activity of PDK, in turn, is controlled by pyruvate, NAD^+^, ATP, acetyl-CoA and nuclear transcription factors such as FoxO, PPAR and PGC1α^15^. From the perspective of the metabolic shift toward glycolysis during OA progression, a potential therapeutic strategy might be derived from metabolic reprogramming to enhance OxPhos through the inhibition of PDK^16^. PDK has four isoforms, PDK1 through PDK4. Among them, PDK2 is expressed ubiquitously, while the remaining isoforms have a tissue-specific distribution: PDK1 is found primarily in heart tissue, pancreatic islets and skeletal muscle; PDK3 has a relatively limited tissue distribution in the testis, kidney and brain; and PDK4 has a relatively limited distribution in the heart, skeletal muscle, liver, kidney and pancreatic islets^17,18^. However, the expression of PDKs in chondrocytes and their functional role in OA have yet to be investigated.

The objective of this study was to determine whether PDK inhibition affects the metabolic flexibility of chondrocytes and contributes to cartilage degeneration in a surgical model of OA. First, we verified the expression of PDK isoforms in both in vitro catabolic conditions and in vivo OA cartilage, which revealed an increase in PDK2 under catabolic conditions. The role of PDK2 in OA progression was investigated via a surgical OA model in *Pdk2*-deficient mice, and we subsequently focused on its metabolic phenotype, expression of anabolic and catabolic factors, reactive oxygen species (ROS) production and cellular senescence. From a mechanistic perspective, we identified the signaling pathways affected by *Pdk2* deficiency and elucidated their role in the production of ROS and cellular senescence.

## Materials and methods

### Ethics and isolation of primary chondrocytes

This study was conducted in accordance with the guidelines of the National Research Council (US) Committee for the Care and Use of Laboratory Animals^19^ and was approved by the institutional review board of the Kyungpook National University School of Medicine (Daegu, Korea) under approval number KNU 2022-0203. Mice that were genetically deficient in the PDK2 gene (PDK2 KO) in the C57BL/6J background were kindly provided by Dr. In-Kyu Lee (Kyungpook National University, Daegu, Korea). Primary chondrocytes were isolated from the articular cartilage of the femurs and tibias of 5-day-old C57BL/6J mice. The isolated cartilage pieces were minced into small fragments, incubated for 15 min in 0.25% trypsin–EDTA at 37 °C with gentle shaking and digested in a solution containing 0.2% collagenase type II, which was prepared in Dulbecco’s modified Eagle medium (DMEM), at 37 °C with gentle agitation for 4 h. After digestion, the cell suspension was filtered through a 40-µm cell strainer and washed twice with phosphate-buffered saline (PBS). The isolated chondrocytes were resuspended in complete 3:2 F12:DMEM-based culture medium supplemented with 0.25% l-glutamine and 0.25% penicillin–streptomycin, seeded in six-well plates at a concentration of 1 × 10^5^ cells per well and incubated at 37 °C in a humidified atmosphere containing 5% CO_2_. To reduce dedifferentiation risk, only chondrocytes at passage 0 were used in the experiments.

### RNA and protein expression analysis

To induce catabolic conditions in primary chondrocytes, the cells were treated with 10 ng/ml IL-1β and incubated for 6 h for RNA isolation and 24 h for protein isolation. For signaling analysis, the cells were starved overnight and then stimulated with 20 ng/ml IL-1β for the indicated times. Total RNA was extracted using TRIzol (Invitrogen), after which first-strand cDNA was generated with Superscript III reverse transcriptase (Invitrogen). A ViiA 7 Real-Time PCR System (Applied Biosystems) and SYBR Green Master Mix (Applied Biosystems) were used for qRT‒PCR. The primers used are listed in Supplementary Table 1. Target gene expression levels were calculated via the 2 − ΔΔCT method and normalized to the geometric mean of GAPDH. All qRT‒PCR analyses were carried out in triplicate and repeated three to five times, and the average results for each sample are presented.

Total proteins were extracted via 300 μl of RIPA buffer supplemented with protease and phosphatase inhibitors (Roche Diagnostics). Total cell lysates containing 10–20 μg protein were subjected to 10% SDS–polyacrylamide gel electrophoresis and transferred onto polyvinylidene difluoride membranes (Immobilon-P; Millipore). The membranes were blocked with 5% skim milk in PBS with 0.25% Tween-20 (PBST) and incubated with primary antibodies against PDK1 (Abcam, #ab202468), PDK2 (#ab68164), PDK3 (#ab154549), PDK4 (#ab214938), phospho-PDH-E1α (Ser232, Millipore, #AP1063), PDH-E1 (Santa Cruz Biotechnology, #sc-377092), Col2 (#ab34712), Sirt1 (Cell Signaling Technology, #9475), MMP13 (#ab51072), p-p38 (#4631), p38 (#9212), p-JNK (#9251), JNK (#9252), p-AMPKα (#2531), AMPKα (#2532), p-FoxO3 (#9466), FoxO3a (#2497), p-mTOR (#2971), mTOR (#2972), p-p65 (#3033), p65 (#8242), p-Akt (#9271), Akt (#9272) and β-actin (Sigma-Aldrich, #A1978) overnight at 4 °C. After being washed with PBST, the membranes were incubated with horseradish-peroxidase-conjugated secondary antibodies at room temperature for 2 h, and then the blots were developed using enhanced chemiluminescence western blotting detection reagent (Thermo Fisher Scientific) and examined via the MicroChemi system (DNR Bio-Imaging Systems). All analyses were performed in biological triplicate, and the western blot band intensities were quantified via ImageJ software (version 1.8.0, National Institutes of Health). The band intensities of the target proteins were normalized to the β-actin band intensity of the respective lanes. The full western blot data are included in Supplementary Fig. 2.

### Surgical OA induction

OA was surgically induced in 12-week-old male C57BL/6J mice, including 7 PDK2 KO mice and 7 of their littermates, through surgical destabilization of the medial meniscus (DMM) under general anesthesia. The sample size of each group was calculated on the basis of the assumptions of a type I error (*α*) of 0.1, a standard deviation (*σ*) of 10, an effect size (*δ*) of 15 and a power of 0.8. The ligaments of the medial meniscus were dissected from the right knee to induce OA, and a sham operation was conducted on the left knee as a control. After surgery, the mice were housed under controlled conditions at 23 ± 1 °C, 50% humidity and a 12-h light/dark cycle. They were kept under specific pathogen-free conditions in the animal facilities of Kyungpook National University Chilgok Hospital.

Eight weeks after surgery, the mice were euthanized via cervical dislocation, the knee joints were collected for histological examination, and all specimens were used for analysis. This study was conducted in accordance with ARRIVE guidelines 2.0, and the checklist is provided in the Supplementary Materials.

### Pain behavior analysis

Beginning 2 weeks after surgery, pain behavior was assessed weekly via the spontaneous weight-bearing asymmetry test (incapacitance test), the hot plate test and threshold punctate mechanical stimulation (the von Frey test). To assess spontaneous weight bearing on the hind limbs, an incapacitance meter (Sang Chung Commercial) was used to measure the downward force applied by each hind limb. The mice were briefly placed in a restraint, with their hind limbs resting on two weight-averaging platform pads. Measurements of the paw pressure of each hind limb were taken for 10 s approximately ten times, and the results were averaged. The data are expressed as the percentage of weight distributed on the ipsilateral hind limb. Next, thermal pain sensitivity was assessed with the hot plate test^20^. The mice were placed on a heated plate (plantar test infrared emitter, Ugo Basile), and the time until they displayed pain behaviors such as licking or shaking their paws was recorded. Finally, tactile allodynia was assessed via the von Frey test. Calibrated monofilaments (von Frey hairs; Stoelting) were applied to the plantar surface of both the ipsilateral and contralateral hind paws, and the mice were placed in an elevated maze in an acrylic cage. Paw withdrawal was considered a positive response. The 50% withdrawal threshold was determined upon six repeated applications of varying force with a von Frey filament via the up–down method^21^.

### Safranin-O and immunofluorescence staining

Mouse knee joints were fixed in 4% paraformaldehyde for 24 h, decalcified in 10% EDTA for 3 weeks and then embedded in paraffin. The embedded blocks were sectioned at a thickness of 6 μm. After deparaffinization and rehydration, the sections were stained with 0.1% Safranin-O solution for 5 min and counterstained with Fast Green solution for 1 min. Cartilage destruction was scored in all four quadrants of the joint (grades 0–24) and on the medial tibial plateau (grades 0–6) by two observers under blinded conditions via the Osteoarthritis Research Society International (OARSI) scoring system^22^. For immunofluorescence staining, rehydrated sections were subjected to antigen retrieval in sodium citrate buffer (10 mM sodium citrate and 0.05% Tween-20, pH 6.0). The sections were then blocked with 2% bovine serum albumin in PBS for 1 h, followed by overnight incubation at 4 °C with primary antibodies against PDK1 (#ab202468), PDK2 (#ab68164), PDK3 (#ab154549), PDK4 (#ab214938), phospho-PDH-E1α (Ser232, #AP1063), PDH-E1 (#sc-377092), MMP13 (#ab51072), 8-oxo-dG (#sc-66036) or normal rabbit IgG in 1% bovine serum albumin. For immunofluorescence, the sections were incubated with Alexa Fluor 488- or 594-conjugated secondary antibodies (Jackson ImmunoResearch Laboratories) for 2 h and then counterstained with 4′,6-diamidino-2-phenylindole (DAPI). Finally, the sections were mounted with anti-fade mounting solution (Vector Labs) and imaged and quantified via a KI-3000F fluorescence microscope.

### Seahorse real-time cell metabolic analysis

Chondrocyte metabolism was analyzed using an XF96 Extracellular Flux Analyzer (Agilent Technologies). Primary chondrocytes from PDK2 KO mice and their littermates were plated in Seahorse XF96 plates at a density of 50,000 cells per well. Confluent chondrocytes were incubated with or without IL-1β (10 ng/ml) for 24 h. For the mitochondrial stress test, the cells were equilibrated for 1 h in serum-free Seahorse XF Base Medium. Basal cellular respiration was initially measured, and mitochondrial respiration inhibitors, including 1 μM oligomycin, 1 μM FCCP and a 1:1 mixture of 2 μM antimycin A with 1 μM rotenone, were sequentially injected into the assay wells. Mitochondrial ATP production was expressed as the oxygen consumption rate (OCR; pmol of O_2_/min). For the glycolysis stress test, the cells were starved for 1 h in glucose-free Seahorse XF DMEM, followed by sequential treatment with 20 mM glucose, 1 μM oligomycin and 100 mM 2-deoxy-D-glucose. Real-time measurements of proton accumulation in the media were taken and quantified as the extracellular acidification rate (ECAR; mpH/min)^23^. After completion of either the mitochondrial or glycolysis stress test, the cell nuclei were counted after in situ DAPI staining. Analysis data were then normalized on the basis of the cell count per well, using a normalization unit of 5,000 cells.

### ATP and NAD^+^/NADH measurements

Chondrocytes from PDK2 KO mice and their littermates were seeded at 5,000 cells per well in 96-well plates for ATP measurement and at 20,000 cells per well in six-well plates for NAD^+^/NADH measurement and treated with or without 10 ng/ml IL-1β for 24 h. The ATP concentration was measured via a commercial ATP detection kit (Abcam, #ab83355) following the manufacturer’s protocol. In brief, chondrocytes were homogenized in 15 μl of ATP assay lysis buffer, and aliquots of the cell lysates were incubated with ATP Reaction Mix for 30 min in the dark. The absorbance of each mixture was quantified via a spectrophotometer at a wavelength of 570 nm. ATP levels were calculated from a standard curve, which was plotted via the serial dilution of authentic ATP.

The NAD^+^/NADH ratio was determined using a commercial NAD^+^/NADH assay kit (Abcam, ab65348) following the manufacturer’s instructions. In brief, chondrocytes were collected by scraping, washed three times with precooled PBS and lysed with extraction buffer solution. After centrifugation at 12,000 rpm for 5 min at 4 °C, the supernatant was collected. The protein concentration was determined via a bicinchoninic acid assay kit (Beyotime Biotechnology). The samples were then heated at 60 °C for 30 min to completely decompose the NAD^+^ in the sample. Then, 50 μl of each sample was mixed with 100 μl of Reaction Mix and incubated at room temperature for 5 min. Subsequently, 10 μl of NADH Developer was added to each well and incubated at room temperature for 2 h. The absorbance of each mixture was quantified via a spectrophotometer at a wavelength of 450 nm.

### Detection of intracellular ROS and oxidative DNA damage in the assessment of oxidative stress

Intracellular ROS and mitochondrial ROS were assessed with dihydroethidium (DHE) and MitoSOX Red (Thermo Fisher Scientific, #M36008) fluorescent dyes, respectively. Primary chondrocytes were fixed with 4% paraformaldehyde for 10 min, permeabilized with 0.25% Triton X-100 and then rinsed three times with PBS. To assess the level of intracellular ROS, the chondrocytes were incubated with 5 μM DHE for 30 min at 37 °C, washed with PBS and then counterstained with DAPI. Images were captured using a KI-3000F fluorescence microscope (Korea Lab Tech).

DNA damage caused by oxidative stress was evaluated by immunofluorescence staining with an 8-oxo-dG antibody. Fixed and permeabilized cells were incubated with the primary 8-oxo-dG antibody overnight at 4 °C. The cells were subsequently incubated with a secondary antibody for 2 h, counterstained with DAPI and imaged using a fluorescence microscope. Fluorescence-positive cells were quantified via ImageJ software.

### Senescence β-galactosidase staining

β-Galactosidase activity was detected using a β-galactosidase staining kit (Cell Signaling Technology, #9860). The cells were fixed with 4% paraformaldehyde for 10 min at room temperature and then incubated for 2 h in staining solution containing 1 mg/ml X-gal, 5 mM potassium ferrocyanide, 5 mM potassium ferricyanide and 2 mM MgCl_2_ in pH 7.4 PBS. Then, the cells were washed with PBS and visualized under a light microscope, and the percentage of blue-colored cells was calculated by counting at least five random fields per sample.

### Statistical analysis

All data are presented herein as the mean ± standard error of the mean (s.e.m.). Statistical analysis to compare the mean values of two groups was performed via the Mann–Whitney *U* test, which is a nonparametric test, because the sample size is small and cannot be assumed to have a normal distribution. *P* values ≤0.05 were considered statistically significant. Statistical analyses were performed with Prism software version 8.0 (GraphPad Software).

## Results

### Among PDK isoforms, PDK2 increases under IL-1β-induced catabolic conditions and in the cartilage of mice with surgically induced OA

**Fig. 1:**
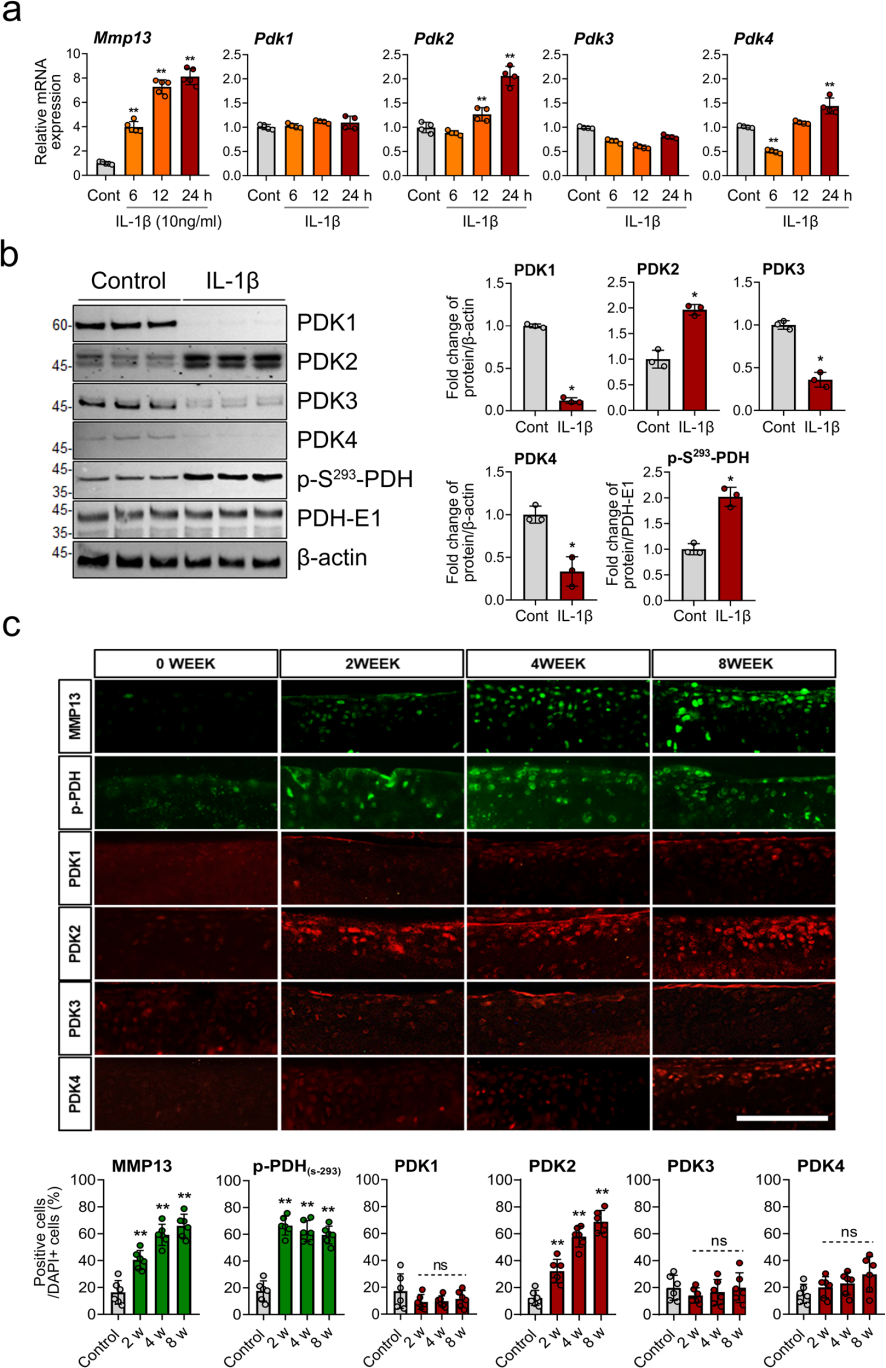
Expression of PDK under IL-1β-induced catabolic conditions and in murine cartilage from surgically induced OA. **a** The expression of *Pdk* isoform (*Pdk1–4*) and *Mmp13* mRNAs in primary chondrocytes at 6, 12 and 24 h after 10 ng/ml IL-1β treatment was assessed via qRT‒PCR. mRNA expression is presented as the fold increase in gene expression normalized to *Gapdh*. **P < 0.01 compared with the PBS control group. Mean ± s.e.m.; Mann–Whitney *U* test; *n* = 4 from biological replicates. **b** After 24 h of treatment with 10 ng/ml IL-1β, the protein levels of the PDK isoforms, phosphorylated PDH (p-S293-PDH) and PDH-E1 in primary chondrocytes were assessed by western blot analysis. Western blot band quantification for PDK1-4 was normalized to β-actin, while that for p-S293-PDH was normalized to PDH-E1. The results are displayed in the form of bar graphs. *P < 0.05 compared with the PBS control group. Mean ± s.e.m.; Mann–Whitney *U* test; *n* = 3 from technical replicates among 2 biological replicates. **c** Immunofluorescence analyses revealed increases in MMP13 and PDK2 in articular chondrocytes at 2, 4 and 8 weeks after DMM surgery. The numbers of MMP13- and PDK isoform-positive chondrocytes above the tidemark were quantified and are expressed as ratios to the number of DAPI-positive cells. Scale bar, 100 μm. ***P < 0.001, compared with control mice. Mean ± s.e.m.; Mann–Whitney *U* test; *n* = 6; ns, not significant.

To understand PDK-mediated metabolic modulation in OA chondrocytes, we first investigated the expression of PDK isoforms under IL-1β-mediated catabolic conditions. Treatment with IL-1β increased the mRNA levels of *PDK2* and *PDK4*, but not *PDK1* or *PDK3*, in primary chondrocytes (Fig. 1a). At the protein level, however, only PDK2 increased after IL-1β treatment. Moreover, the level of the phosphorylated, inactive form of PDH (p-S^293^-PDH) increased, suggesting that PDK2 may be involved in the phosphorylation of PDH under IL-1β-mediated catabolic conditions (Fig. 1b). To confirm this increase in PDK2, we examined the expression of PDK isoforms in the articular cartilage over time after DMM surgery in mice. Phosphorylated PDH, which indicates the inactivation of PDH, increased at 2 weeks post-DMM surgery. This increase was accompanied by an increase in PDK2, which continued to increase over time. PDK4 tended to increase at approximately 8 weeks, but this increase was not statistically significant, whereas PDK1 and PDK3 did not increase in the DMM-induced model of OA (Fig. 1c).

### PDK2 deficiency attenuates the severity of OA, oxidative stress and pain-related behaviors in a DMM-induced murine OA model

**Fig. 2:**
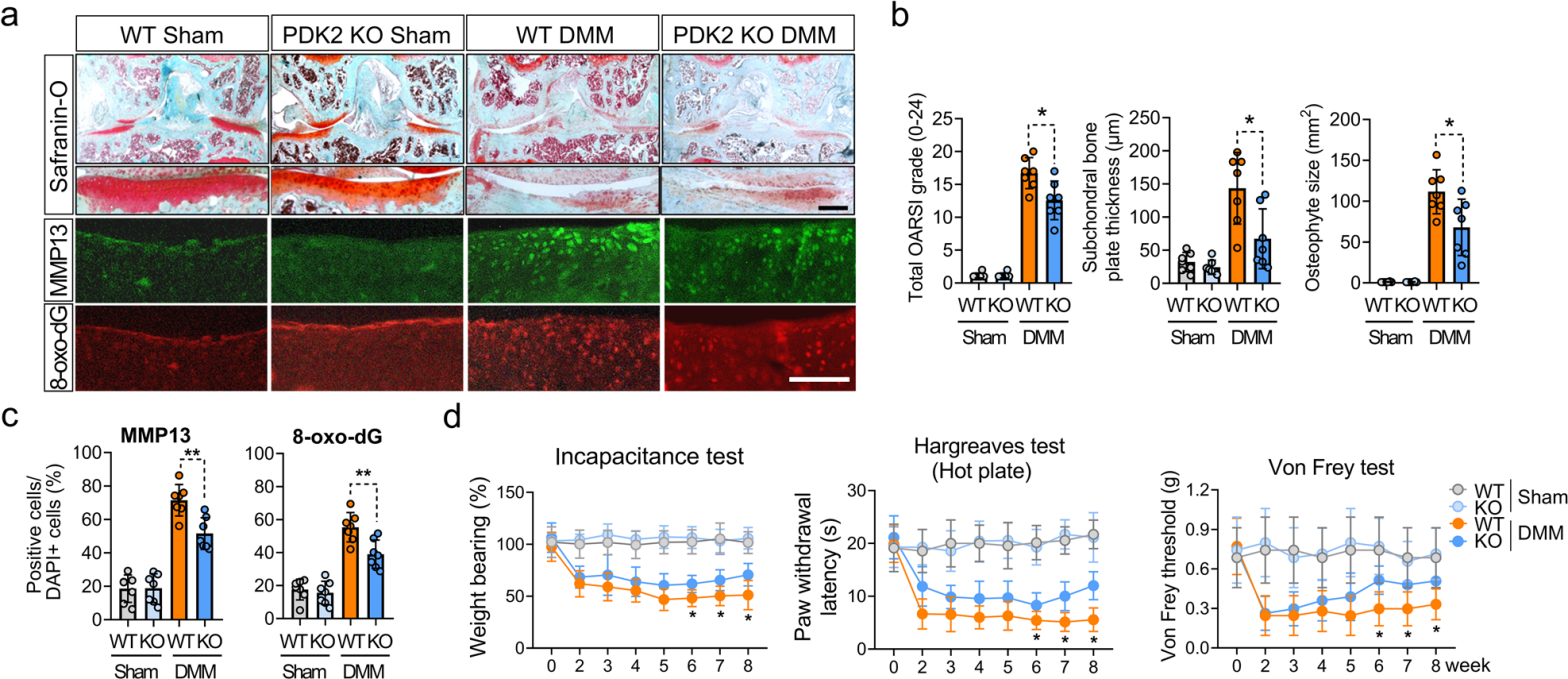
PDK2 deficiency reduces the severity of cartilage degradation, oxidative stress-related DNA damage and pain-related behaviors in DMM-induced OA mice. **a** Safranin-O staining and immunostaining for MMP13 and 8-oxo-dG were performed on WT and *Pdk2* KO mice 8 weeks after DMM and sham surgery, and representative images are displayed. Scale bars, 100 μm. **b** OARSI grade, subchondral bone plate thickness and osteophyte size were quantified and are expressed as mean ± s.e.m. *P < 0.05, Mann–Whitney *U* test. *n* = 7. **c** Quantification of the percentage of MMP13- and 8-oxo-dG-positive chondrocytes above the tidemark. ***P < 0.001; mean ± s.e.m.; Mann–Whitney *U* test; *n* = 7. **d** Pain behavior tests were conducted once a week on DMM and sham-operated mice. Weight bearing on the hind paw was assessed by an incapacitance test, which represents the ratio of weight bearing between the ipsilateral and contralateral hind paws; thus, any percentage less than 100% indicates hind limb unweighting. Thermal and mechanical pain sensations were assessed with the Hargreaves test via a hot plate and the von Frey test, respectively. *P < 0.05, compared between WT and *Pdk2* KO mice in the DMM group, Mann–Whitney *U* test. *n* = 7.

To assess the impact of chondrocyte metabolic reprogramming on OA progression, we compared the phenotypes of DMM-induced OA in wild-type (WT) and *Pdk2* KO mice. Compared with WT mice, genetic deletion of PDK2 significantly decreased the progression of DMM-induced OA. This was indicated by the decrease in cartilage degradation, as quantified by the OARSI score, and reduced osteophyte maturation and subchondral bone thickness (Fig. 2a, b and Supplementary Fig. 1). This finding was further supported by immunofluorescence staining, which revealed fewer MMP13-positive and 8-oxo-dG-positive chondrocytes in *Pdk2* KO mice than in control mice, indicating a decrease in the levels of ECM-degrading proteases and oxidative stress-induced DNA damage, respectively (Fig. 2a, c). This effect was accompanied by decreased pain-related behaviors in *Pdk2* KO mice compared with WT mice beginning at 6 weeks post-DMM surgery, as observed in tests such as static weight bearing over the hind limbs (incapacitance test), paw withdrawal time on a hot plate, and the von Frey test (Fig. 2d).

### PDK2 deficiency partially restores the IL-1β-mediated metabolic shift toward glycolysis in chondrocytes

**Fig. 3:**
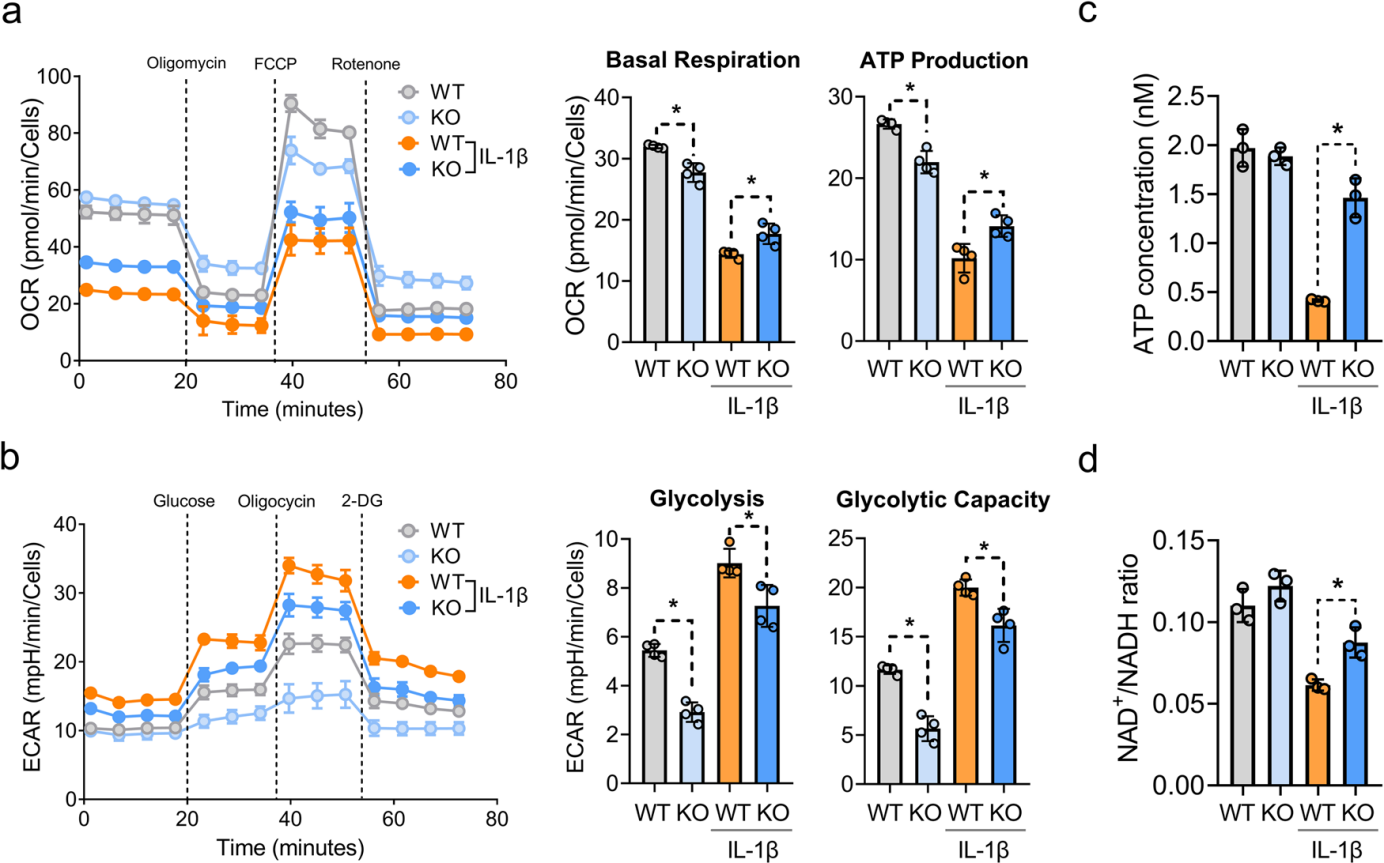
PDK2 deficiency partially restores IL-1β-induced metabolic alterations, leading to increases in ATP and NAD^+^/NADH levels in primary chondrocytes. **a**,**b**, OCRs (**a**) and ECARs (**b**) were measured in primary chondrocytes isolated from WT and *Pdk2* KO mice via an XF96 Seahorse analyzer. Basal respiration and ATP production in the OCR analysis and glycolysis and glycolytic capacity in the ECAR analysis at one time point were quantified and are presented as the mean ± s.e.m. *P < 0.05, Mann–Whitney *U* test. *n* = 4. **c**, **d**, The ATP levels (**c**) and NAD^+^/NADH ratios (**d**) in the culture media were quantified via colorimetric assays at 570 nm and 450 nm, respectively; mean ± s.e.m., *P < 0.05, Mann–Whitney *U* test. *n* = 3.

To determine whether PDK2 deficiency impacts chondrocyte metabolism, we assessed OxPhos and glycolysis under IL-1β-induced catabolic conditions using the Seahorse XF96 Extracellular Flux Analyzer. IL-1β treatment for 24 h markedly suppressed OxPhos, as reflected by a decrease in the OCR of cultured primary chondrocytes, whereas it increased glycolysis, as indicated by an increased ECAR. PDK2 deficiency partially restored the OCR by increasing basal respiration and ATP production in chondrocytes under IL-1β-treated conditions (Fig. 3a). Meanwhile, the IL-1β-mediated increase in the ECAR was significantly reduced in *Pdk2-*deficient chondrocytes, resulting from decreased glycolysis and glycolytic capacity (Fig. 3b). This PDK2-mediated metabolic reprogramming also led to a significant increase in ATP and the NAD^+^/NADH ratio in the culture supernatant from *Pdk2-*deficient chondrocytes (Fig. 3c, d). These findings suggest that the inhibition of PDK2 enhances OxPhos, potentially restoring the balance of energy homeostasis in chondrocytes under catabolic conditions such as those in OA.

### PDK2 deficiency enhances PDH activity and anabolic effects, which lead to reduced oxidative stress and cellular senescence in chondrocytes under IL-1β-treated conditions

**Fig. 4:**
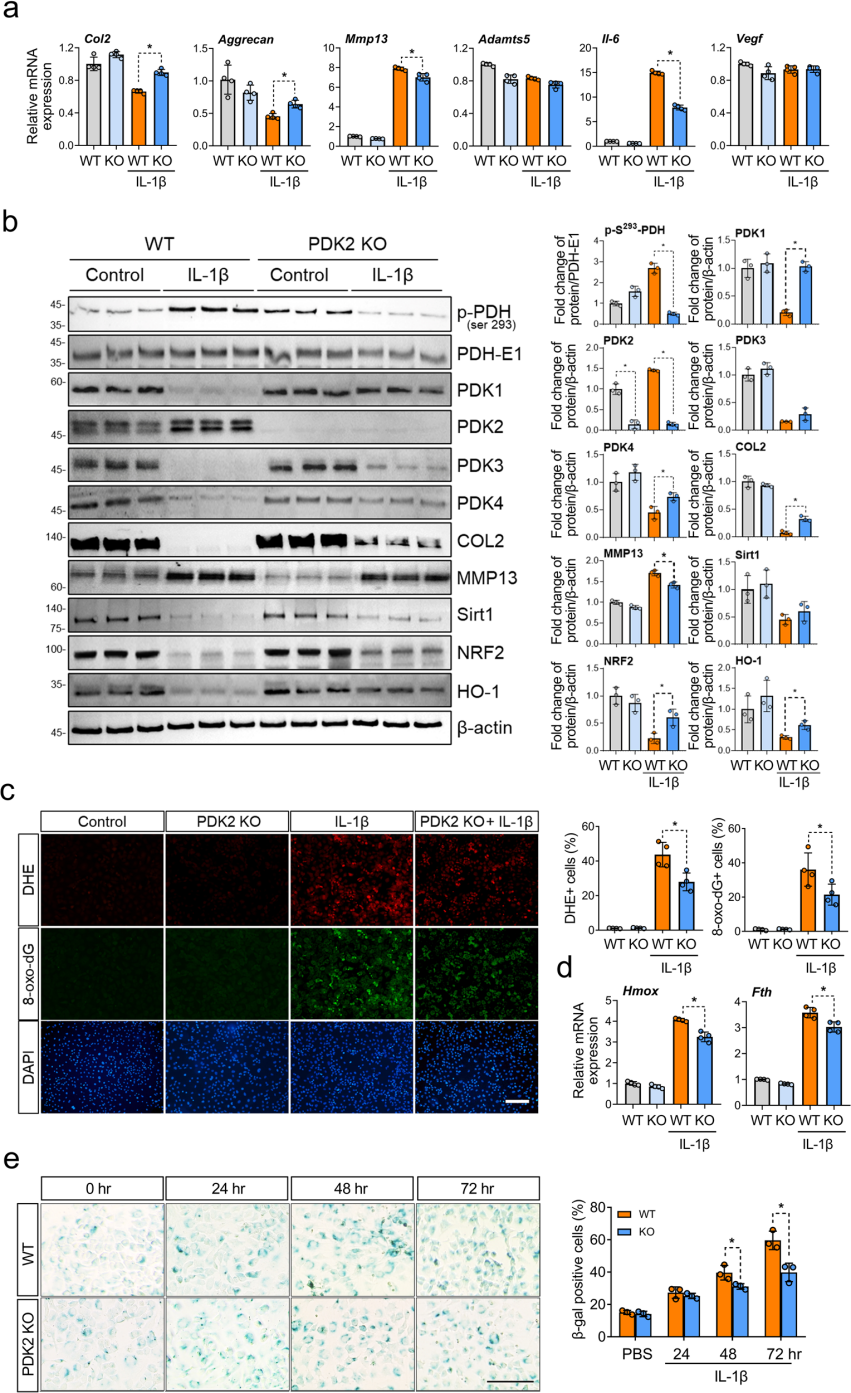
PDK2 deficiency not only increases the active form of PDH and has anabolic effects on IL-1β-treated chondrocytes but also decreases oxidative stress and cellular senescence. **a** The expression of chondrogenic marker genes such as *Col2* and *Aggrecan*, catabolic proteases such as *Mmp13* and *Adamts5*, and SASP-related biomarkers such as *Il-6* and *Vegf* was assessed via qRT‒PCR. The results for mRNA expression are displayed as the fold increase in gene expression normalized to *Gapdh*. Mean ± s.e.m., *P < 0.05, Mann–Whitney *U* test. *n* = 4. **b** The phosphorylated-PDH (p-S^293^-PDH), PDH-E1, PDK1, PDK2, PDK3, PDK4, Col2, MMP13, Sirt1, NRF2 and HO-1 protein levels in WT and *Pdk2* KO chondrocytes after 24 h of IL-1β were assessed by western blot analysis. Western blot band quantification for p-S^293^-PDH protein levels was normalized to PDH-E1, and the levels of PDK1, PDK2, PDK3, PDK4, Col2, MMP13, Sirt1, NRF2 and HO-1 were normalized to those of β-actin. *P < 0.05, Mann–Whitney *U* test. *n* = 3. **c** Staining with DHE (red), a fluorescent probe for ROS, and 8-oxo-dG (green), a marker for oxidative DNA damage, was conducted in primary chondrocytes from WT and *Pdk2* KO mice treated with IL-1β (10 ng/ml) for 24 h. The nuclei of the cells were counterstained with DAPI (blue). Scale bar, 100 μm. DHE and 8-oxo-dG fluorescence-positive cells were quantified and expressed as ratios to DAPI-positive cells. *P < 0.05. Mann–Whitney *U* test. *n* = 4. **d** The relative expression of oxidative stress marker genes such as *Hmox* and *Fth* was evaluated via qRT‒PCR. The mRNA expression results are displayed as the fold increase in gene expression normalized to *Gapdh*. *P < 0.05. Mann–Whitney *U* test. *n* = 4. **e** SA-β-gal staining was conducted on primary chondrocytes treated with IL-1β (10 ng/ml) for the indicated times. Scale bar, 100 μm. β-Gal-positive cells were counted from six different fields from three biological replicates, and the percentages of positive cells were determined. *P < 0.05. Mann–Whitney *U* test. *n* = 3.

To investigate the impact of PDK2 deficiency on chondrocyte homeostasis under catabolic conditions, we then examined the mRNA expression of chondrogenic markers (*Col2* and *Aggrecan*), catabolic proteases (*Mmp13* and *Adamts5*) and senescence-associated secretory phenotype (SASP)-related genes (*Il-6* and *Vegf*). Under IL-1β-treated conditions, PDK2 deficiency increased *Col2* and *Aggrecan* expression and decreased *MMP13* and *IL-6* expression but had no effect on *Adamts5* or *Vegf* expression (Fig. 4a). PDH enzymatic activity was enhanced in *Pdk2*-deficient chondrocytes, as indicated by lower levels of phosphorylated PDH (p-S^293^-PDH) under IL-1β-treated conditions. PDK2 deficiency significantly increased the protein levels of PDK1 and PDK4 under IL-1β-treated catabolic conditions but not under untreated control conditions, suggesting compensatory upregulation of other PDK isoforms. Similarly, the protein levels of Col2 increased in PDK2-deficient chondrocytes compared with WT chondrocytes under IL-1β-treated conditions, whereas those of MMP13 decreased. The protein levels of Sirt1 remained unaffected, but the levels of antioxidant proteins, such as NRF2 and HO-1, were increased in *Pdk2*-deficient chondrocytes under IL-1β-treated conditions (Fig. 4b). Next, we assessed the role of PDK2 in oxidative stress under IL-1β-treated catabolic conditions. IL-1β treatment (10 ng/ml) significantly increased the production of ROS as well as 8-oxo-dG, a DNA damage product that occurs as a result of oxidative stress. Under these conditions, *Pdk2*-deficient chondrocytes presented a significant decrease in ROS and 8-oxo-dG production, suggesting that PDK2 can reduce oxidative stress under IL-1β-mediated catabolic conditions (Fig. 4c). Consistent with these results, the expression of *Hmox* and *Fth*, which are target genes of oxidative stress, was decreased in *Pdk2*-deficient chondrocytes (Fig. 4d). We further investigated the effect of PDK2 deficiency on the senescence of chondrocytes, as mitochondrial dysfunction and ROS production are closely related to cellular senescence^24^. After 48 h of treatment with IL-1β, a marked decrease in the expression of senescence-associated β-galactosidase (SA-β-gal), a senescence marker, was observed in chondrocytes from PDK2 KO mice compared with those from WT mice (Fig. 4e). Our results collectively indicate that PDK2 loss of function leads to anabolic effects and attenuates ROS levels as well as the senescence of chondrocytes under catabolic conditions.

### PDK2 deficiency reduces p38 signaling activity and sustains AMPK activation in response to IL-1β stimulation

**Fig. 5:**
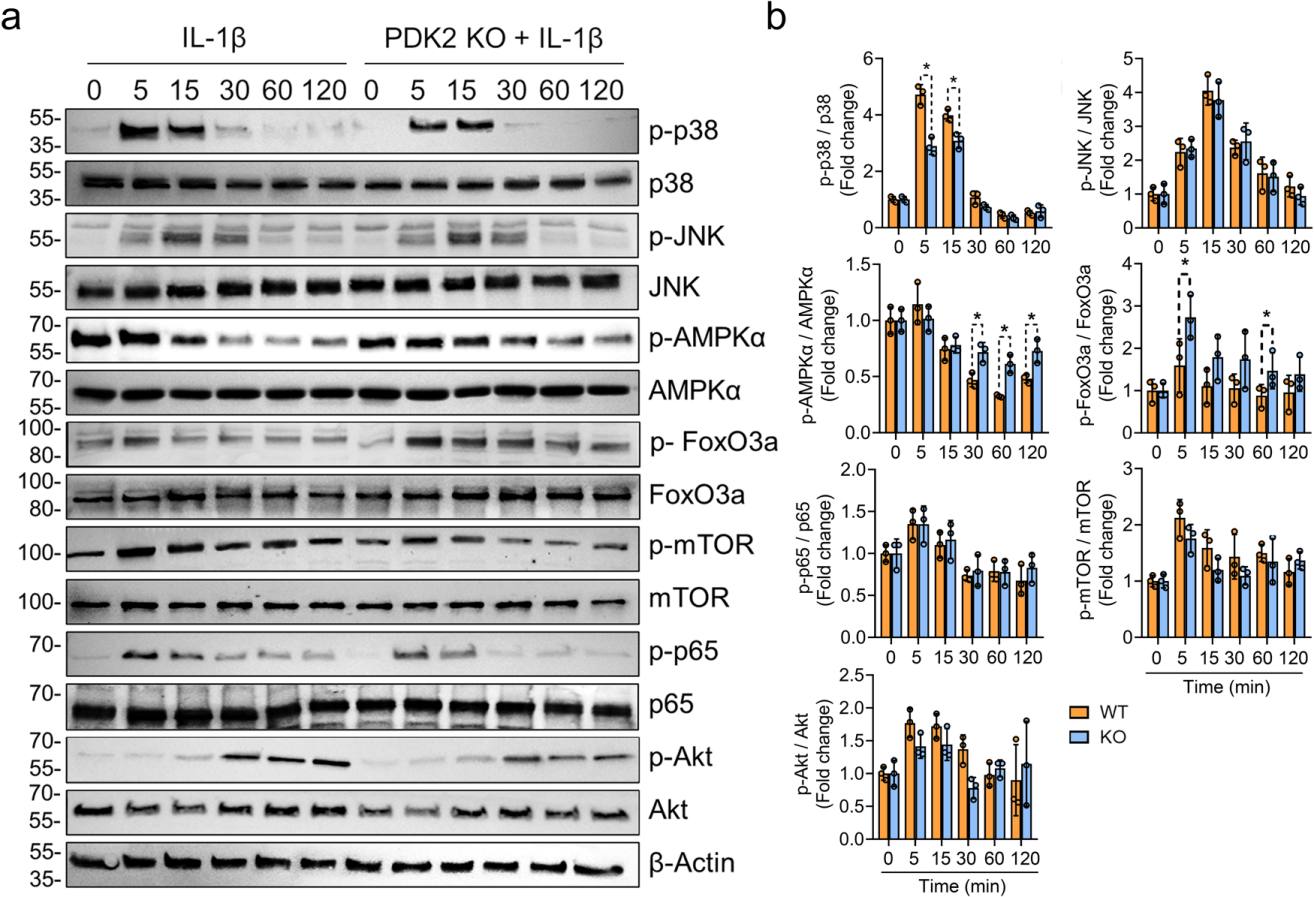
PDK2 deficiency enhances FoxO3a signaling and prevents the downregulation of AMPK signaling, while it suppresses p38 MAPK activity under IL-1β-induced catabolic conditions. **a** The phosphorylation of FoxO3a, AMPKα, p38, JNK, mTOR, p65 and Akt was assessed in primary chondrocytes from WT and Pdk2 KO mice stimulated with IL-1β (20 ng/ml) for the indicated durations. The experiments were performed in triplicate, and representative blots are shown. **b** The western blot band quantification for phosphorylated proteins was based on normalization to the corresponding nonphosphorylated total protein. *P < 0.05. Mann–Whitney *U* test. *n* = 3.

We further examined the signaling mechanisms involved in the anabolic effects on PDK2-deficient chondrocytes under catabolic conditions, with a particular focus on metabolism and oxidative stress-related pathways such as the AMPK, mTOR, FoxO3a, AKT, NF-κB and MAPK pathways. Among these signaling pathways, the phosphorylation of p38 MAPK (Thr180/Tyr182) was most prominently suppressed in *Pdk2*-deficient chondrocytes compared with WT chondrocytes. The phosphorylation of AMPK (Thr172), which is typically downregulated by IL-1β stimulation, remained in an activated state in *Pdk2*-null chondrocytes. PDK2 deficiency led to a discontinuous increase in FoxO3a phosphorylation (Ser253) at 5 and 60 min after IL-1β stimulation, which suppressed FoxO3a activity through cytoplasmic export and proteasomal degradation^25^. The activation of mTOR, JNK, p65 and AKT signaling was not affected by PDK2 deficiency (Fig. 5a, b).

### p38 MAPK is essential for generating ROS in chondrocytes with mitochondrial dysfunction, and mitochondrial ROS, in turn, activate p38 MAPK under catabolic conditions

**Fig. 6:**
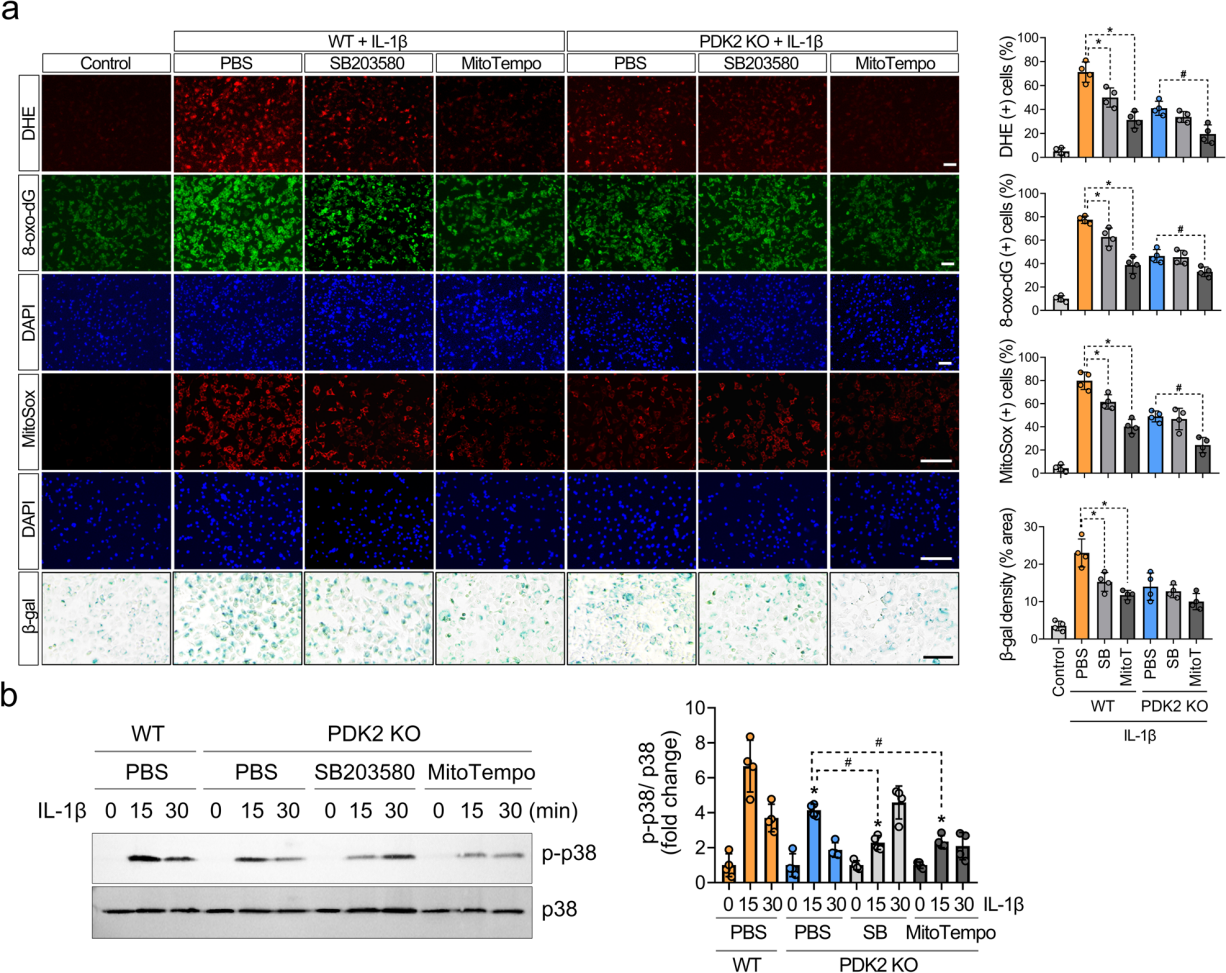
PDK2 is involved in a positive feedback loop between p38 MAPK and mitochondrial ROS production. **a** p38 MAPK inhibition led to a decrease in ROS production and cellular senescence in WT chondrocytes but not in *Pdk2*-deficient chondrocytes. Chondrocytes from WT and *Pdk2* KO plants were pretreated with the p38 MAPK inhibitor SB203580 and the mitochondrial ROS inhibitor Mito-TEMPO for 30 min. The cells were then treated with IL-1β (10 ng/ml) for 24 h and stained with DHE (red) for total cellular ROS, 8-oxo-dG (green) for DNA damage, and MitoSOX (red) for mitochondrial ROS, and their nuclei were counterstained with DAPI (blue). Cellular senescence was assessed via SA-β-gal staining. Scale bar, 100 μm. DHE, 8-oxo-dG and MitoSOX fluorescence-positive cells were quantified and expressed as ratios to DAPI-positive cells. The area of SA-β-gal expression was quantified via densitometry with ImageJ software. *P < 0.05, ^#^*P* < 0.05. Mann–Whitney *U* test. *n* = 4. **b** p38 MAPK phosphorylation was assessed in the presence of a p38 inhibitor (SB203580) and a mitochondrial ROS inhibitor (Mito-TEMPO). Chondrocytes from WT and *Pdk2* KO mice were stimulated with IL-1β (20 ng/ml) for the indicated times and subjected to western blot analysis to assess the phosphorylated and total protein levels of p38 MAPK. *P < 0.05 compared with the fold change in p-p38/p38 in WT PBS controls at 15 min after stimulation. ^#^*P* < 0.05 compared with p-p38/p38 in *Pdk2* KO PBS controls at 15 min after stimulation. Mann–Whitney *U* test. *n* = 4.

To determine whether the decrease in p38 MAPK phosphorylation is simply a result of reduced ROS or if it directly involves a reduction in ROS in PDK2-deficient conditions, we treated WT and *Pdk2*-deficient chondrocytes with the chemical p38 inhibitor SB203580 and the specific scavenger of mitochondrial superoxide Mito-TEMPO. The inhibition of p38 significantly suppressed ROS production and cellular senescence in WT chondrocytes, but this effect was not observed in *Pdk2*-deficient chondrocytes, where ROS generation and cellular senescence remained unchanged despite p38 MAPK inhibition (Fig. 6a). This finding implies that, in a metabolic environment in which OxPhos is increased due to PDK2 deficiency, p38 MAPK does not play a significant role in ROS production and subsequent cellular senescence.

Next, we investigated how the inhibition of mitochondrial ROS affects the activation of p38 MAPK. PDK2 deficiency led to a significant reduction in the phosphorylation of p38 MAPK compared with that in chondrocytes from WT littermates under IL-1β stimulation, and treatment with SB203580 substantially delayed the activation of p38 MAPK in *Pdk2*-deficient chondrocytes. As expected, scavenging mitochondrial superoxide via Mito-TEMPO significantly reduced the phosphorylation of p38 MAPK at 15 min compared with that of both the WT and *Pdk2*-deficient controls, indicating that mitochondrial ROS are crucial for the activation of p38 (Fig. 6b). Collectively, these results suggest the presence of a positive feedback loop between ROS and p38 MAPK and highlight the significant role of the PDK-mediated glycolytic metabolic shift in p38 MAPK-mediated ROS generation.

## Discussion

**Fig. 7:**
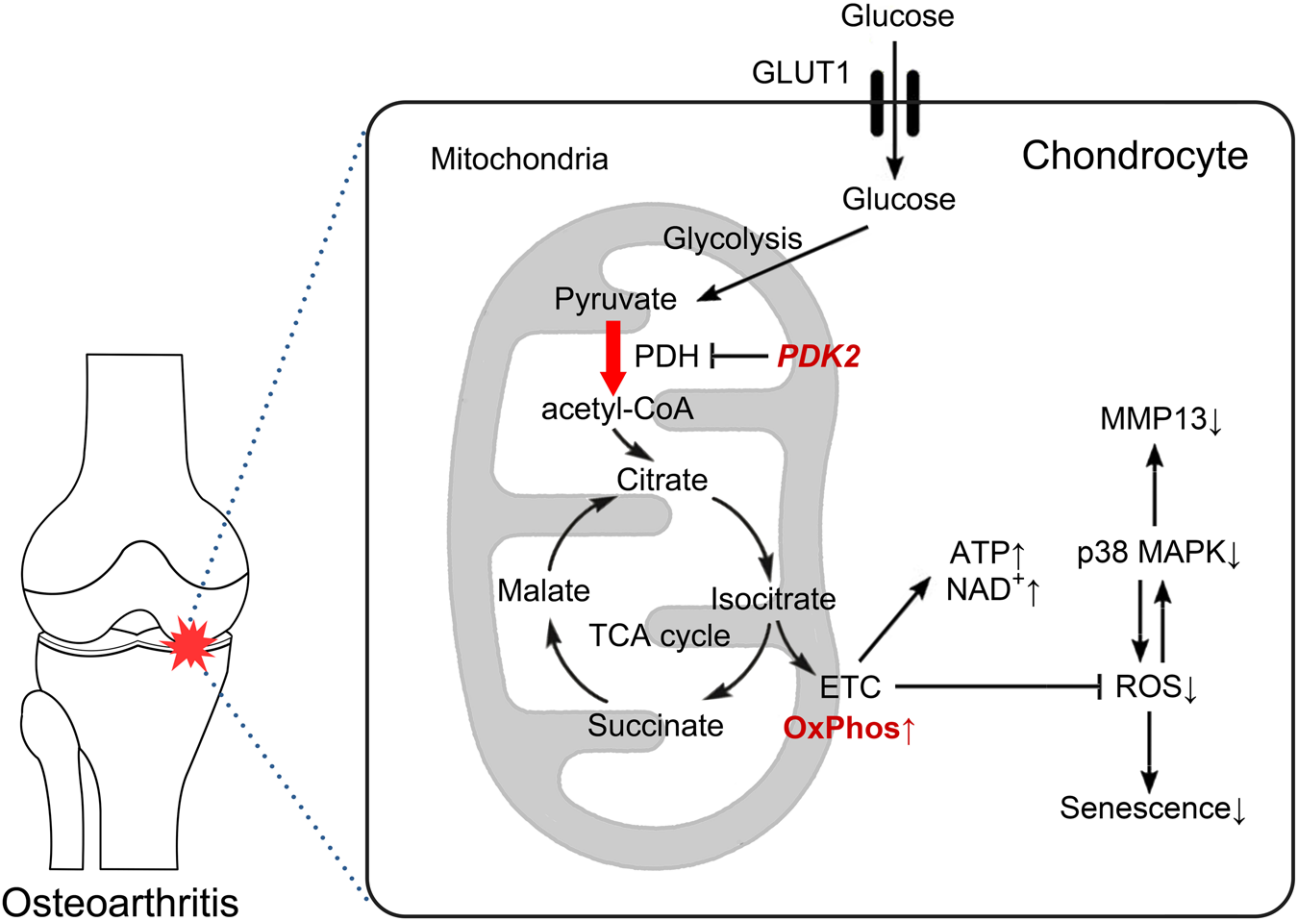
Schematic diagram depicting the anabolic effects of metabolic reprogramming toward oxidative phosphorylation caused by PDK2 deficiency under catabolic conditions. Among PDK isoforms, PDK2 was expressed primarily under catabolic and in vivo OA conditions. The loss of PDK2 function enhanced OxPhos and ATP/NAD^+^ production, which led to a reduction in oxidative stress. Mechanistically, PDK2 plays a crucial role in the positive feedback loop between oxidative stress and p38 MAPK under catabolic conditions in chondrocytes.

Increasing evidence suggests that alterations in chondrocyte metabolism toward glycolysis, associated with mitochondrial dysfunction, are critically linked to OA pathogenesis^8,9^. In this context, PDK-dependent inhibition of PDH activity may be a pivotal mechanism responsible for the glycolytic metabolic shift in catabolic chondrocytes^26^. Here, we revealed that PDK2 is specifically upregulated in OA chondrocytes and that its loss of function leads to an increase in PDH activity in order to restore the IL-1β-mediated metabolic shift toward glycolysis in chondrocytes. In addition, PDK2 deficiency resulted in a protective effect in a surgically induced OA model, which was accompanied by reduced oxidative stress and cellular senescence. Mechanistically, PDK2 deficiency led to decreased activation of p38 MAPK, along with sustained activation of AMPK signaling under IL-1β-treated conditions (Fig. 7). Taken together, our data shed light on the potential of metabolic reprogramming toward OxPhos as a novel therapeutic approach for OA.

Several lines of evidence indicate the critical involvement of mitochondrial dysfunction in the pathogenesis of OA^9,27^. Specifically, chondrocytes from patients with advanced OA exhibit a decrease in respiring mitochondria, as indicated by decreased rhodamine 123 staining^9^. Morphologically, these mitochondria are characterized by increased length relative to width, coupled with an overall reduction in count and disrupted morphology^9^. This elongation is particularly noteworthy, as it implies increased mitochondrial fusion, a phenomenon often observed under conditions such as nutrient withdrawal or increased OxPhos^28,29^. Chondrocytes from relatively preserved articular cartilage demonstrate significantly greater mitochondrial respiration capacity than those from severely damaged lesions do^9^. However, as OA progresses, these metabolic adaptations begin to fail. This is indicated by a diminished capacity of the respiratory chain and by a decrease in the number of mitochondria coupled with an increase in mitochondrial fission, leading to mitochondrial dysfunction^9,27^. Consequently, impaired mitochondrial function can disrupt ATP production and increase oxidative stress in chondrocytes, both of which are key contributors to the pathogenesis of OA^30^.

From this perspective, the central hypothesis of this study was that enhancing OxPhos could inhibit the progression of OA. This study revealed that PDK2 deficiency, along with the subsequent increase in PDH activity, increased OxPhos under catabolic conditions, leading to a reduction in oxidative stress and the inhibition of OA progression. These results conflict with the general consensus that increasing OxPhos leads to an increase in ROS^31^. However, a line of evidence has shown that inhibiting mitochondrial respiration can actually increase ROS production^32–35^. Taken together, these findings suggest that both excessive and defective mitochondrial respiration can contribute to increased ROS production, depending on the cellular context. Our in vitro experiments revealed that treatment with dichloroacetate, which is a pan-PDK inhibitor that induces a switch in cellular metabolism toward OxPhos, increased ROS production in nonsenescent chondrocytes but decreased ROS in H2O2- and IL-1β-induced senescent chondrocytes (Supplementary Fig. 3). This result suggests a context-dependent role of OxPhos in ROS production; under physiological conditions, an increase in OxPhos increases ROS, but under stress or senescent conditions, an increase in OxPhos may conversely reduce ROS production. Our data suggest that restoring OxPhos in OA chondrocytes is a promising approach to reduce ROS and the catabolic cascade, thereby potentially inhibiting the progression of OA.

So far, the molecular mechanism underlying the metabolic shift toward glycolysis in OA chondrocytes has remained largely unclear. In this study, we demonstrated a significant increase in PDK2 among PDK isoforms under IL-1β-mediated catabolic conditions and in OA chondrocytes (Fig. 1). Moreover, PDK2 deficiency led to a decrease in the phosphorylation of PDH under IL-1β-treated catabolic conditions, indicating inactivation of the PDH complex, which converts pyruvate to acetyl-CoA (Fig. 4b). These findings suggest that PDK2 may be a key regulator of chondrocyte metabolism under catabolic conditions. Indeed, our data confirmed that PDK2 deficiency, at least partially, enhanced OxPhos in IL-1β-treated chondrocytes while reducing glycolysis (Fig. 3). PDK, which serves as a negative feedback mechanism, is activated by the products of the PDH reaction and the TCA cycle, such as NADH, high energy charge and acetyl-CoA^17^. This activation leads to inactivation of the PDH. However, a decreasing energy charge and increasing pyruvate concentration inhibit PDK activity, thereby leading to increased PDH activation^36^. Although several mechanisms, including lactate dehydrogenase-A, hypoxia-inducible factor 1A, the AKT–mTOR signaling pathway and pyruvate kinase M2, are known to control the glycolytic shift in chondrocytes^8,37–39^, PDK is known to mediate the Warburg effect, which is characterized by increased aerobic glycolysis^40^. This may directly lead to the glycolytic shift observed in OA. Given these findings, inhibiting PDK2 could be a promising approach for the metabolic reprogramming of chondrocytes.

The expression of PDK isoforms in chondrocytes has not been extensively characterized. Our data revealed an increase in PDK2 and a decrease in other PDK isoforms, such as PDK1, PDK3 and PDK4, in IL-1β-treated catabolic chondrocytes and in vivo OA cartilage (Fig. 1). Consistent with our findings, a recent study reported significant downregulation of PDK1 mRNA and protein expression in OA articular cartilage, although it did not specify the expression of other PDK isoforms^41^. The lack of any noticeable phenotype in endochondral bone formation in *Pdk2*-deficient mice also suggests that PDK2 plays a limited role in the physiological maturation of chondrocytes (data not shown). This evidence that PDK2 is specifically expressed in catabolic chondrocytes suggests that targeting PDK2 could minimally affect normal cartilage physiology while effectively addressing OA conditions, thereby offering a potential advantage in the development of OA drugs targeting PDK2.

Our data revealed that IL-1β-mediated p38 MAPK phosphorylation was significantly reduced in *Pdk2*-deficient chondrocytes (Fig. 5). Apoptosis signal-regulating kinase 1, which is positioned upstream of p38 MAPK, is a well-known redox-sensitive kinase^42,43^, implying that lower ROS levels in *Pdk2*-deficient chondrocytes may lead to reduced phosphorylation of p38 MAPK. Furthermore, p38 MAPK signaling itself can induce oxidative stress via MAP kinase-activated protein kinase 2, potentially creating a positive feedback loop between p38 MAPK and oxidative stress under catabolic conditions^44^. Furthermore, our data revealed that the p38 inhibitor significantly suppressed ROS generation in WT chondrocytes, whereas this inhibition of ROS was not observed in PDK2 KO chondrocytes (Fig. 6a). This finding implies that p38 MAPK does not influence ROS generation in conditions prone to OxPhos due to PDK2 deficiency. In other words, p38 MAPK may primarily contribute to an increase in ROS in situations of mitochondrial dysfunction, characterized by reduced OxPhos. Although OA is primarily a degenerative disease, omics data from OA articular cartilage have revealed a sustained increase in inflammatory signatures^45,46^. Our findings indicate that p38 MAPK could be an essential intermediary linking metabolic alterations to the inflammatory gene signature in OA cartilage.

Another significant observation regarding signaling changes associated with PDK2 deficiency is the more gradual reduction in AMPK phosphorylation (Thr172) caused by IL-1β stimulation. As implied by its name ‘AMP-activated protein kinase’, AMPK is activated by AMP, which typically increases under metabolic stress conditions^47^. Conversely, when metabolic balance is restored and ATP levels rise, this leads to inactivation of the kinase^48^. In addition to metabolic conditions, the regulation of AMPK involves several upstream kinases; LKB1, CaMKKβ and TAK1 are key activators, whereas PKC, AKT, PKA and PP2A contribute to its inactivation^49^. With respect to oxidative stress, although AMPK activation helps suppress it, oxidative stress can, in turn, lead to the inactivation of AMPK^50^. Thus, the reduced levels of ROS observed in PDK2 deficiency could slow the inactivation of AMPK, potentially aiding in the maintenance of metabolic homeostasis in chondrocytes under catabolic conditions.

In conclusion, the loss of function of PDK2, which is upregulated under catabolic conditions in chondrocytes, leads to a metabolic shift toward OxPhos. This shift is associated with a reduction in oxidative stress and cellular senescence, providing protective effects against OA progression. Our findings suggest that metabolic modulation toward OxPhos deserves particular attention as a potential target for OA treatment.

## Supplementary information

Supplementary Information

## Data Availability

The datasets used and/or analyzed during the current study are available from the corresponding author upon reasonable request.
