# Supplementary material for: Increased oxidative phosphorylation through pyruvate dehydrogenase kinase 2 deficiency ameliorates cartilage degradation in mice with surgically induced osteoarthritis
Source: Exp Mol Med. 2025 Feb 3;57(2):390–401. doi: 10.1038/s12276-025-01400-9 (PMC11873213; doi:10.1038/s12276-025-01400-9)

Supplementary Table1. Primer List for qPCR Analysis

Gene	Primer	Primersequences
PDK1	forward	CCGGGCCAGGTGGACTTC
	reverse	GCAATCTTGTCGCAGAAACATAAA
PDK2	forward	GCTTCCCCTGACCTGGAGAT
	reverse	AGGCTGGACTCGGCTTT
PDK3	forward	CGGTCCCCAAGCAGATCGA
	reverse	GTTAGCCAGTCGCACAGGAG
PDK4	forward	CACATGCTCTTCGAACTCTTCAAG
	reverse	TGATTGTAAGGTCTTCTTTCCCAAG
Vegf	forward	GAGAGAGGCCGAAGTCCTTT
	reverse	TTGGAACCGGCATCTTTATC
Col2	forward	GGCAATAGCAGGTTCACGTACA
	reverse	GATAACAGTCTTGCCCCACTTACC
Hmox	forward	TGACCACGTGACCAACTTACG
	reverse	CCAGGGTGTGCTTGTCAAAGA
Txn	forward	ATCAAGCCCTTCTTCCATTCC
	reverse	TCCTGGCAGTCATCCACATC
Arg	forward	ACATCAACACTCCCCTGACAA
	reverse	TACGTCTCGCAAGCCAATGTA
Adamts5	forward	CCATCTTCCCGGTTGTGTATCT
	reverse	ACTGTTTCACTCTGGGCTCTT
MMP13	forward	GGACTCACTGTTGGTCCCTG
	reverse	GGATTCCCGCAAGAGTCACA
IL6	forward	CCGGAGAGGAGACTTCACAG
	reverse	TCCACGATTTCCCAGAGAAC
Gapdh	forward	AGCCCAAGATGCCCTTCAGT
	reverse	CCGTGTTCCTACCCCCAATG

Supplementary Fig. 1. Raw data Safranin-O staining of Figure 2a

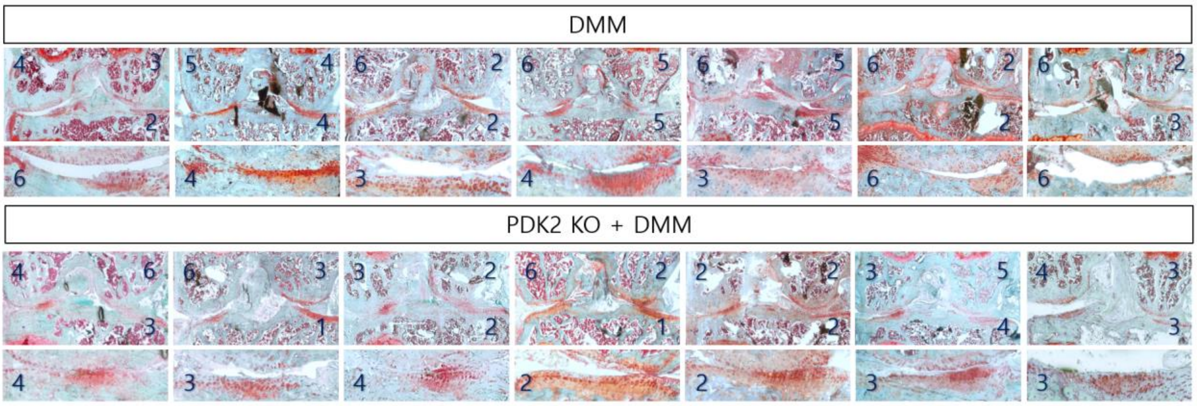

Supplementary Fig. 2. Raw data of Western blot of this manuscript

Figure 1b

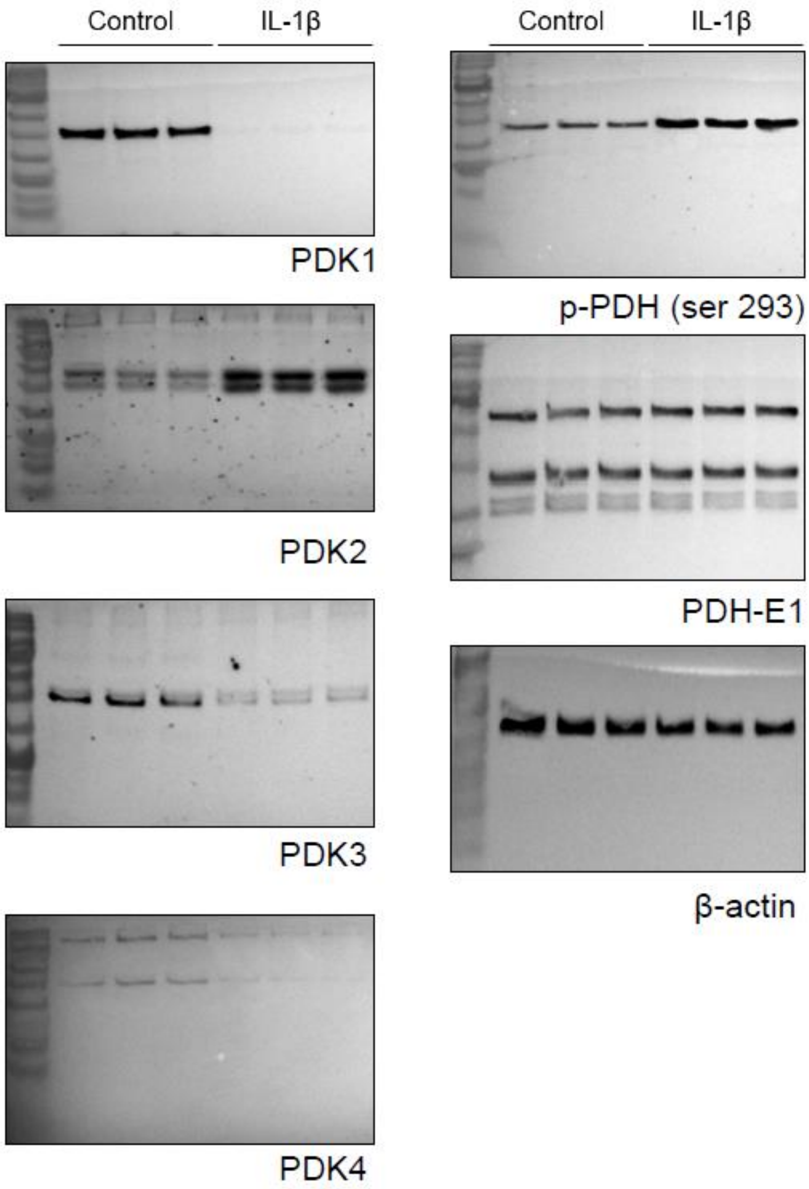

Figure 4b

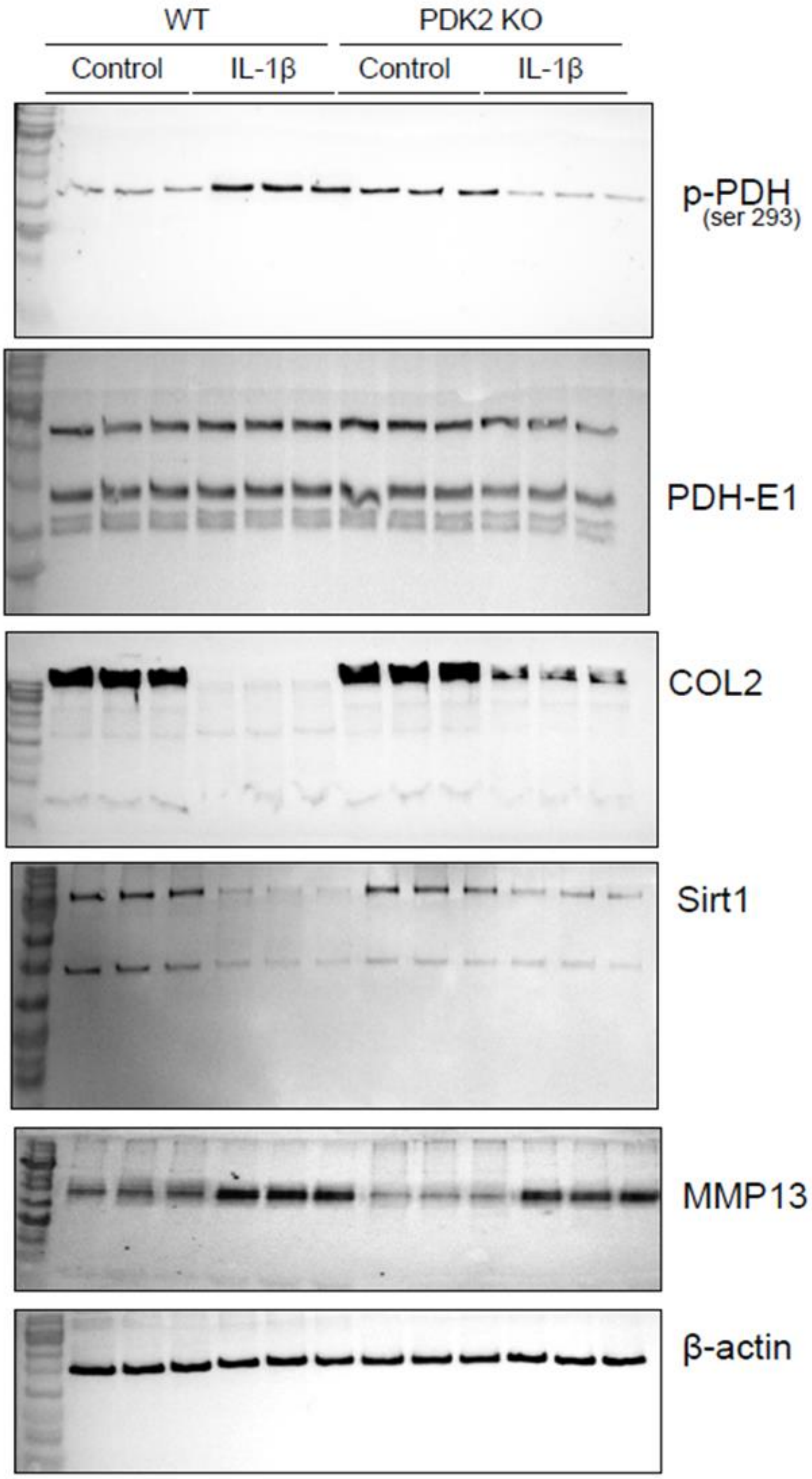
WT
PDK2 KO
Control
IL-1β
Control
IL-1β
p-PDH (ser 293)
PDH-E1
COL2
Sirt1
MMP13
β-actin

Figure 5a-1

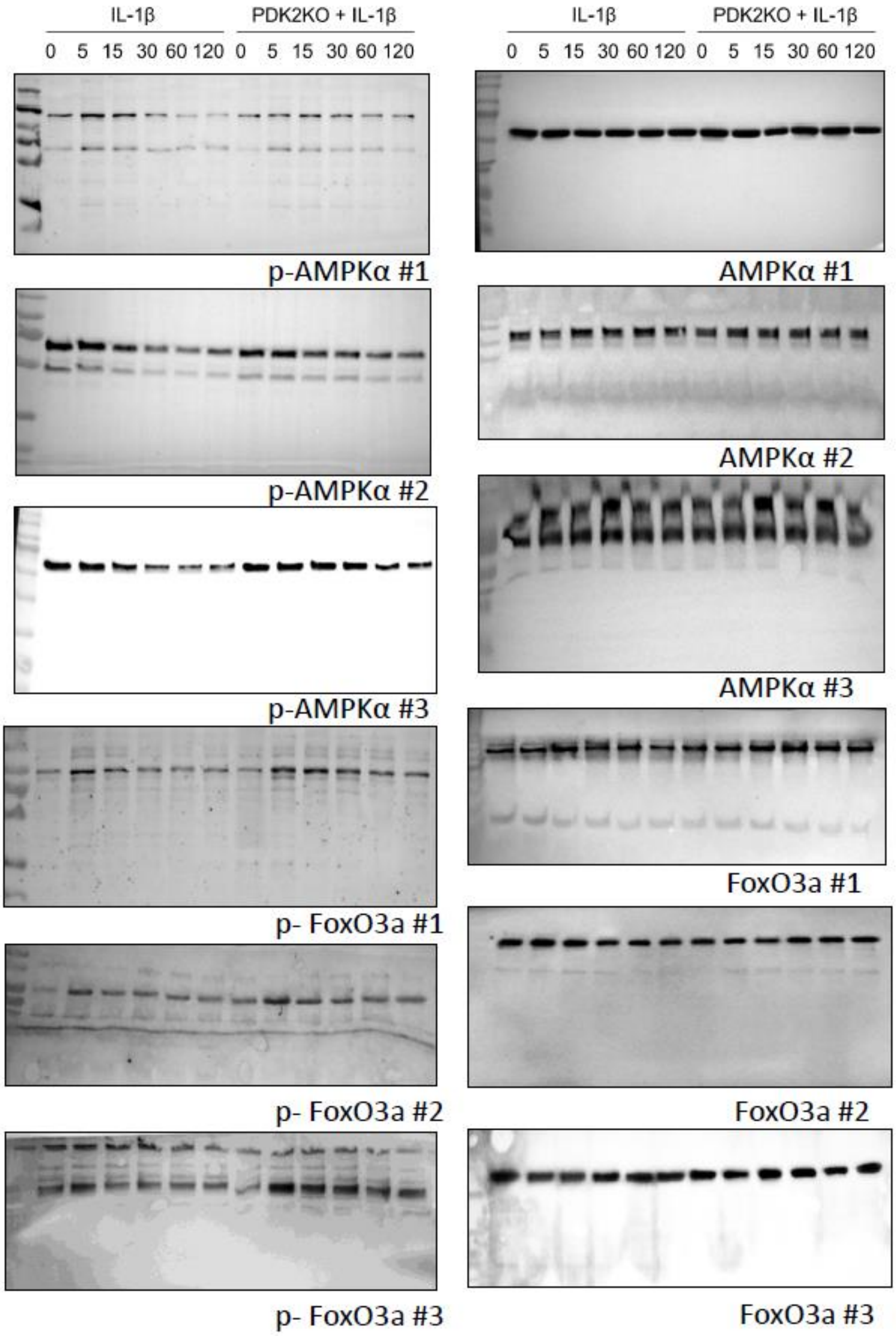
IL-1β
PDK2KO + IL-1β
0 5 15 30 60 120 0 5 15 30 60 120
IL-1β
PDK2KO + IL-1β
0 5 15 30 60 120 0 5 15 30 60 120
p-AMPKα #1
AMPKα #1
p-AMPKα #2
AMPKα #2
p-AMPKα #3
AMPKα #3
p- FoxO3a #1
FoxO3a #1
p- FoxO3a #2
FoxO3a #2
p- FoxO3a #3
FoxO3a #3

Figure 5a-2

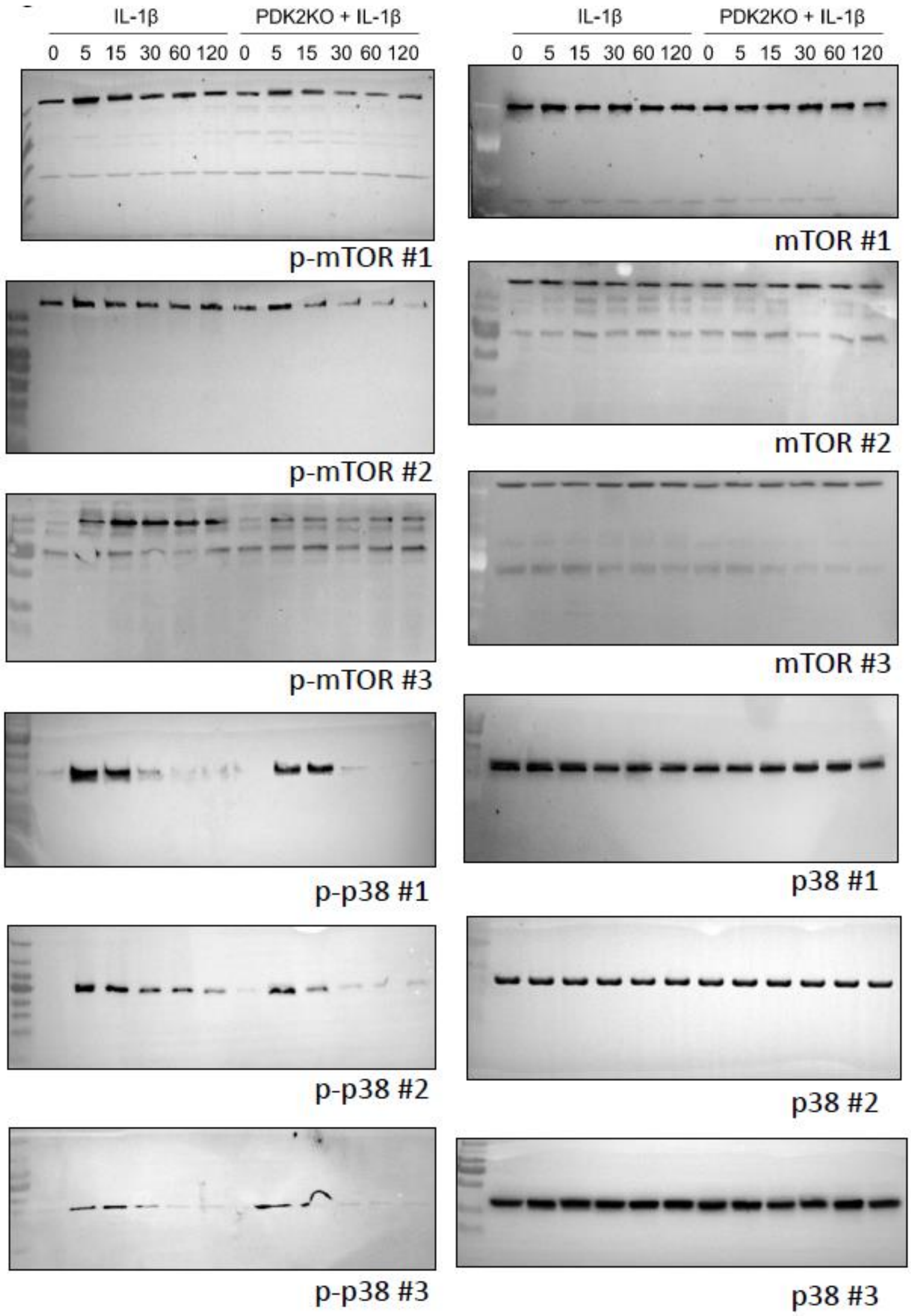
IL-1β
PDK2KO + IL-1β
0 5 15 30 60 120 0 5 15 30 60 120
IL-1β
PDK2KO + IL-1β
0 5 15 30 60 120 0 5 15 30 60 120
p-mTOR #1
mTOR #1
p-mTOR #2
mTOR #2
p-mTOR #3
mTOR #3
p-p38 #1
p38 #1
p-p38 #2
p38 #2
p-p38 #3
p38 #3

Figure 5a-3

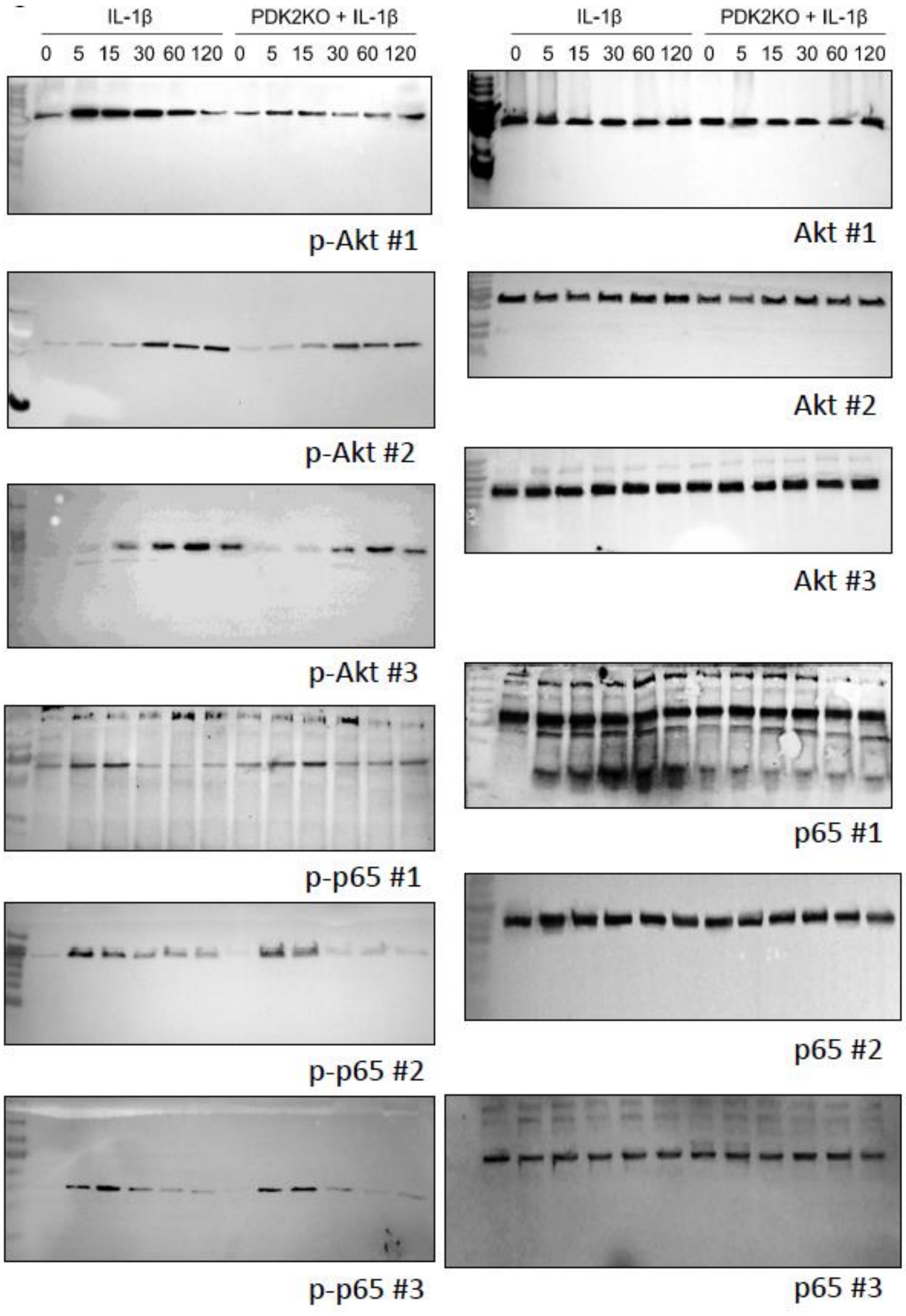
IL-1β
PDK2KO + IL-1β
0 5 15 30 60 120 0 5 15 30 60 120
IL-1β
PDK2KO + IL-1β
0 5 15 30 60 120 0 5 15 30 60 120
p-Akt #1
Akt #1
p-Akt #2
Akt #2
p-Akt #3
Akt #3
p-p65 #1
p65 #1
p-p65 #2
p65 #2
p-p65 #3
p65 #3

Figure 5a-4

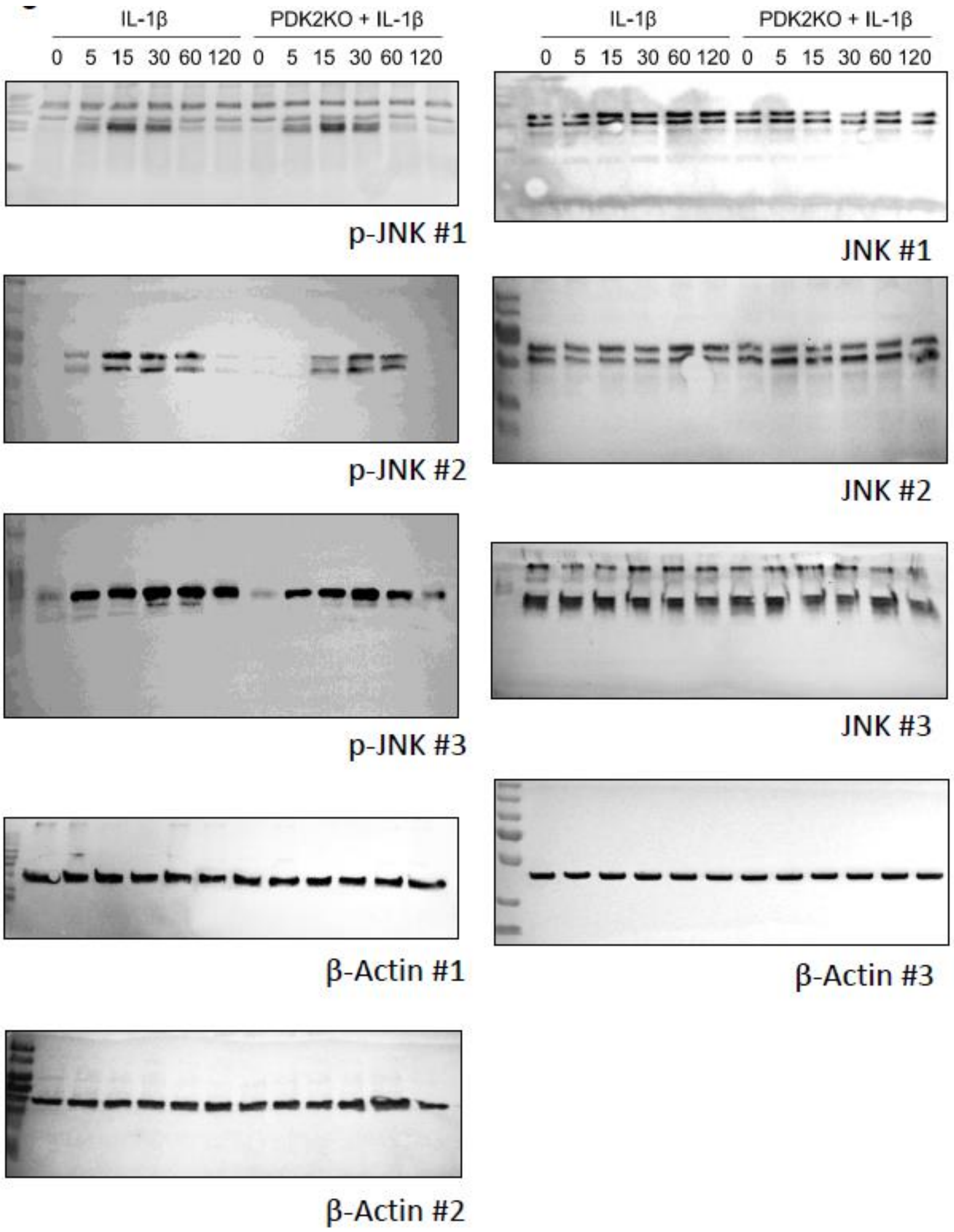

IL-1β
PDK2KO + IL-1β
0 5 15 30 60 120 0 5 15 30 60 120
IL-1β
PDK2KO + IL-1β
0 5 15 30 60 120 0 5 15 30 60 120
p-JNK #1
JNK #1
p-JNK #2
JNK #2
p-JNK #3
JNK #3
β-Actin #1
β-Actin #3
β-Actin #2

Figure 6b

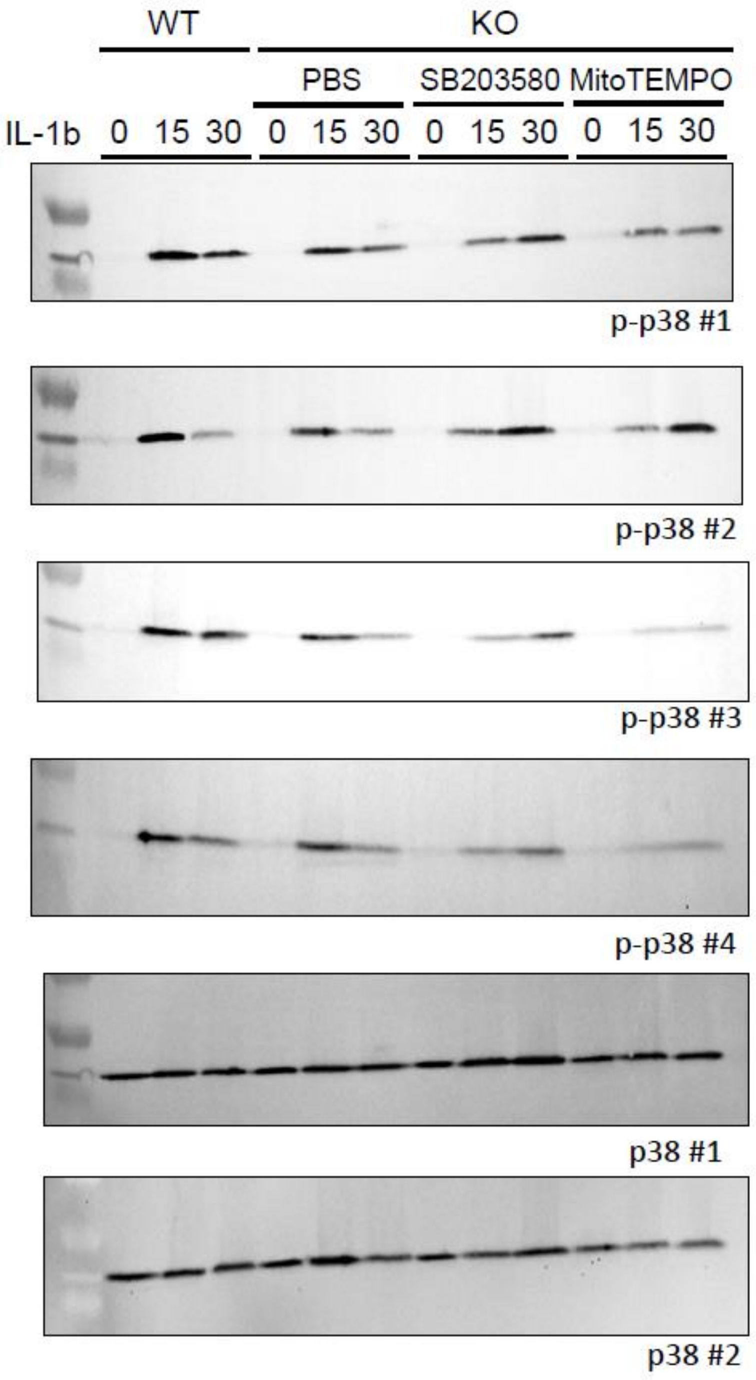
WT
KO
PBS
SB203580
MitoTEMPO
IL-1b 0 15 30 0 15 30 0 15 30 0 15 30
p-p38 #1
p-p38 #2
p-p38 #3
p-p38 #4
p38 #1
p38 #2

Supplementary Fig. 3. The effects of dichloroacetate (DCA), a pan-NOX inhibitor, on ROS and senescence in primary chondrocytes are context-dependent.

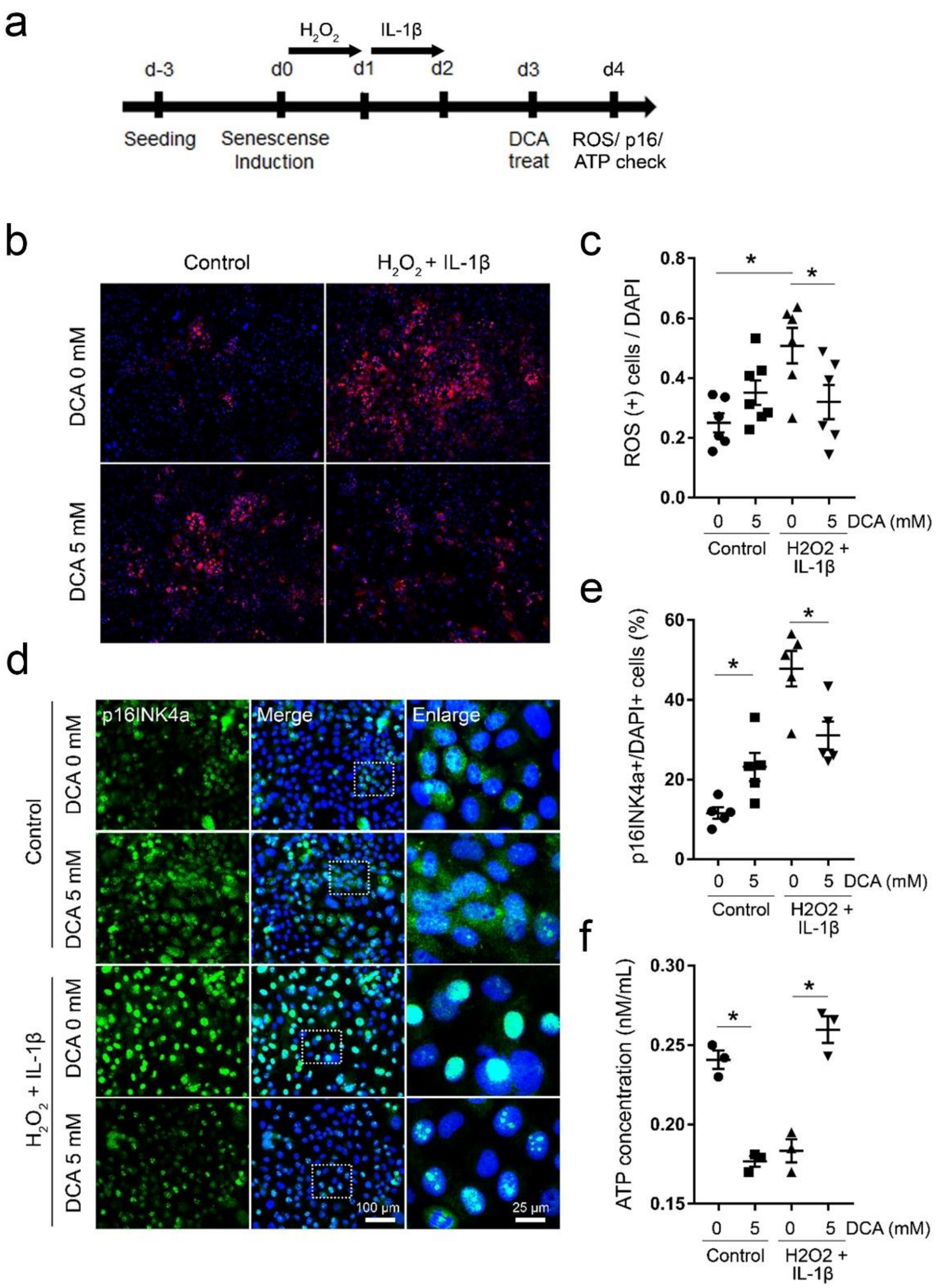

Supplement: Supplementary file 1 — Supplementary Information [file 12276_2025_1400_MOESM1_ESM.pdf]
